# Event and Comment

**Published:** 1914-02-15

**Authors:** 


### DENTAL REGISTER.

**Vol. LXVIII. February 15, 1914. No. 2**

### EVENT AND COMMENT.

STRIKE OF THE GERMAN DENTAL STUDENTS. Through the kindness of Dr. Eugene F. Strom of Landau, Germany, we present the cause of the recent trouble among the dental schools of Germany.

There is a considerable dissatisfaction among German dentists because no title or degree is given for the long and exacting college curriculum. There is a class of dentists called "Technickers" who have had no college training, who in reality under present laws can practice all the technical features of dentistry except the surgical and therapeutic department; and dentists from other countries holding degrees are permitted to practice in the country even in the surgical and therapeutic departments. The suggestion has been advanced that the dentists take the medical degree or degrees in phylosophy, etc. But of course this would greatly extend the time required and increase the cost of the course to such an extent that few could afford to take the course. The educational authorities don't care to add another degree, such for instance as Doctor of Dental Surgery, for which the dental students have petitioned, and there is a dead lock, and the cause of the strike. It would seem from the report published below in the Frankfurter Zeitung of January 4th, that there is now a disposition on the part of the Universities to grant the degree, if the Prussian Educational Department consents.

The demand of the Zahnarzts is that dentistry be recognized as a distinct profession, and that a doctorate degree shall be given for the completion of a reasonable course of study in a standard German University.

January 3rd, 1914, a large gathering of the dentists of Germany met to consider the demands made by dental students for the Degree of Doctor of Dental Surgery, conducted by Prof. Dr. Sachs of Berlin. A number of University professors of the Dental and Medical Faculties of Berlin and Munchen, conducted under the chairmanship of Dr. Sachs and the chairman of the Joint Society of German Dentists, the President of the Prussian Dental Society, Dr. Sheele of Kassel, the chairman of all the Dental Societies of Berlin and nearly all Germany, were represented at the meeting. The chairman of the meeting emphasized that it was immaterial whether an M. D., a D. D. S., or a Dr. of the Science of Dentistry should be established. However, we should avoid demanding an M. D., degree under special examination requirements, which no doubt woul bring about the degrading of the dental profession, as it would be secondary to the regular Medical Degree. A promotion by the Medical Faculty, even under foreign requirements, is in no way ideal as the already expensive study of the science of dentistry would be made even more expensive and more lengthy by adding the study for the Medical Degree, consequently there would surely be a decline in the growth of German educated dentists more pronounced than ever before.

Dr. Frohman of Berlin advocated as first representative, the doctorate of dentistry, in preference to the long and expensive study of general medicine and the interest of dental science. Of course General Medical Science is fundamental as an aid to the science of dentistry, but it is not essential. Dental technique is a science, which needs any number of aids, such as Metallurgy, Physics, Chemistry, Anatomy, etc. Experience has taught the necessity of an independent study of the science of dentistry, which could not possibly be submerged in one of its aiding sciences, as the sad experiment in Oesterreich has shown. The close relationship of dentistry to all its aiding sciences develops innumerable problems, which can only be solved by dental specialists.

Dr. Bolton of Husum, discussed largely questions of social position and the situation according to the National-protection-regulations. He said the strike of the students was a last cry for help. They returned to the lecture-rooms, as soon as they were promised a friendly consideration of their petitions.

Professor Walkhoff of Munchen strengthened what had been said by the other representatives, by citing experiences he had had in his long career as a University professor.

A resolution was then unanimously adopted which very strongly advocated the petition for a promotion of dentistry and its recognition as a distinct profession. The strike of the students demonstrated that the preparatory study of dentistry for a full-academy course was no longer desirable under the present conditions. The people would suffer for lack of educated dentists throught this. In addition to this the difficult social situation of the dentist in comparison with the class of techincers. The present protection-regulations classes the tooth-technicker with those who are lawfully entitled to practice dental surgery, so that the difference between a man who passes the state board examination and a technicker with but a public school education is not known to the general public. As the number of dental students becomes smaller, the development of the science of dentistry, as well as the perfecting of its technic, and the good of the people through lack of scientifically trained dentists will suffer very much. In addition to this the great work our educated German Dentists are doing in the development of the science of dentistry has been most successful and fully accredited by all foreign countries, who would soon take advantage of our failure to supply our own needs by sending their graduates to us. It would be the biggest mistake to undertake to solve the doctor question by merging the art and science of dentistry with general medicine, as the development of dentistry in other countries abundantly shows.

On the statement of the Rector of the University of

Berlin that the Prussian Minister of Education would confer with a representative committee from the several national dental schools and professional bodies in regard to the matter, the striking students have returned to their classes. As the profession at large and a large majority of the Universities favor granting the doctorate degree it seems likely that some favorable action will be taken at a near date.

NEW FEATURES IN DENTAL JOURNALS FOR 1914. The *Dental Cosmos* comes out with a new "front" this year, which is neat and good to look at, the same buff color and material but a stronger paper and more simple type selection. All in very good taste.

The *Pacific Gazette* also spring a new front of orange colored paper with a two tone ink-print. The design is artistic and symbolic of California, as it contains a picture of the Old Mission Church at San Diego, with a border of the state flower, the yellow poppy,—inclosing the seal of the Panama Pacific Congress. While the design is rather conspicuous it has a real significance and is pleasant to contemplate from our more severe climate. The *Items of Interest* has made a complete change in its pictorial heads of Departments; beginning with a guardian Angel defending its "Exclusive Contributions," and closing with a much more pleasing "Roman Herald," who supplants the former lagubrious prolocutor, who has for so long presided over the department of Society Announcements. We notice the headings are all copyrighted, we are wondering why! The Editor has started a new department called "The Round Table," in which he threatens to report the comments made about authors of papers read and discussed in open meetings of dental societies, by the timid fellows around the tables, and *sub rosa* as it were, after the meeting is over.

This will be interesting but it is a hazardous venture which may unless judiciously edited have its come backs!

DENTAL HYGIENISTS. Dr. A. C. Fones is putting into practice his conviction that there is need of better educated and trained office assistants, by inaugurating a training school in the city of Bridgeport, Connecticut. His method is to have a series of lectures given by men who are interested and capable of speaking authoritatively on the several phases of general and special hygiene. He has in training in the present class some 32 women who will take up the work of assistants in dental offices on a more scientific basis and with better preparation than has heretofore been customary. Something like twenty lectures on various corelated topics will be given, illustrated as fully as is practicable to make them comprehensible. Afterwards a six weeks course in technical training will be given the students to prepare them for active duty. It certainly is a most creditable venture and we can't help admiring the man who not only had convictions, but will make the personal sacrifice to demonstrate his ideas. We can't say that the name dental hygienest is wholly satisfactory, but it is so much more acceptable than the "dental nurse," formally suggested, that we shall gladly accept it if the experiment is conclusive. There can be little doubt of the necessity for some addition to our professional resources and it may be that this scheme will supply the temporary demand at least. There is so much that is hopeful in the direction of oral and dental prophylaxis at the present time, from the active scientific agitation of the subject that we are hoping that the future has in store for us less of surgical and more of scientific therapeusis for preventive dentistry. Until this comes however, we must do the best possible with the present situation. Dr. Fones may be starting a practicable solution.

DENTISTS HONORED. The dentists of Wisconsin tendered a banquet to Doctors Arthur Holbrook and B. G. Maerklein, Wednesday, January 21st at the Hotel Pfister in Wilwaukee, who have recently retired from practice. Dr. Holbrook graduated from the Philadelphia Dental College in 1867, and Dr. Maerklein from the University of Pennsylvania in 1886. These men have contributed much to the progress of the profession in Wisconsin and elsewhere, and it is highly important that some appreciation of their unselfish endeavors should be recognized in this public manner.

---

Dr. B. J. Cigrand, the versatile editor of the American Dental Journal has again surprised his friends by being appointed by the Governor of Illinois to the important position of State Director of Brokerage Loans. Now we don't know just what this means or why the Governor of Illinois selected Dr. Cigrand for this office. A very flattering editorial comment appears in the Western Trade Journal which is the official organ of financial affairs published in Chicago. We don't know whether Cigrand is to control the loan sharks or the banks of Chicago and Illinois: in either case he will tackle a big job, but from our knowledge of the man he will meet the situation with energy and a training of broad experience.

